# Automatic Pest Counting from Pheromone Trap Images Using Deep Learning Object Detectors for *Matsucoccus thunbergianae* Monitoring

**DOI:** 10.3390/insects12040342

**Published:** 2021-04-12

**Authors:** Suk-Ju Hong, Il Nam, Sang-Yeon Kim, Eungchan Kim, Chang-Hyup Lee, Sebeom Ahn, Il-Kwon Park, Ghiseok Kim

**Affiliations:** 1 Department of Biosystems Engineering, College of Agriculture and Life Sciences, Seoul National University, 1 Gwanak-ro, Gwanak-gu, Seoul 08826, Korea; hsj5596@snu.ac.kr (S.-J.H.); yskra@snu.ac.kr (S.-Y.K.); oxycle@snu.ac.kr (E.K.); dlckdguq731@snu.ac.kr (C.-H.L.); three97@snu.ac.kr (S.A.); 2 Department of Agriculture, Forestry and Bioresources, College of Agriculture and Life Sciences, Seoul National University, Seoul 08826, Korea; skadlfeksl@snu.ac.kr (I.N.); parkik1@snu.ac.kr (I.-K.P.); 3 Global Smart Farm Convergence Major, College of Agriculture and Life Sciences, Seoul National University, 1 Gwanak-ro, Gwanak-gu, Seoul 08826, Korea; 4 Research Institute of Agriculture and Life Science, College of Agriculture and Life Sciences, Seoul National University, Seoul 08826, Korea

**Keywords:** pest monitoring, sex pheromone trap, *Matsucoccus thunbergianae*, deep learning, CNN, object detection, Faster R-CNN, SSD, insect counting

## Abstract

**Simple Summary:**

The black pine bast scale, *Matsucoccus thunbergianae*, is a forest pest that causes widespread damage to black pine; therefore, monitoring this pest is necessary to minimize environmental and economic losses in forests. However, monitoring insects in pheromone traps performed by humans is labor intensive and time consuming. To develop an automated monitoring system, we aimed to develop algorithms that detect and count *M. thunbergianae* from images of pheromone traps using deep-learning-based object detection algorithms. Object detection models based on deep learning neural networks under various conditions were trained, and the performances of detection and counting were compared and evaluated. In addition, the models were trained to detect small objects well by cropping images into multiple windows. As a result, the algorithms based on deep learning neural networks successfully detected and counted *M. thunbergianae*. These results showed that accurate and constant pest monitoring is possible using the artificial-intelligence-based methods we proposed.

**Abstract:**

The black pine bast scale, *M. thunbergianae*, is a major insect pest of black pine and causes serious environmental and economic losses in forests. Therefore, it is essential to monitor the occurrence and population of *M. thunbergianae*, and a monitoring method using a pheromone trap is commonly employed. Because the counting of insects performed by humans in these pheromone traps is labor intensive and time consuming, this study proposes automated deep learning counting algorithms using pheromone trap images. The pheromone traps collected in the field were photographed in the laboratory, and the images were used for training, validation, and testing of the detection models. In addition, the image cropping method was applied for the successful detection of small objects in the image, considering the small size of *M. thunbergianae* in trap images. The detection and counting performance were evaluated and compared for a total of 16 models under eight model conditions and two cropping conditions, and a counting accuracy of 95% or more was shown in most models. This result shows that the artificial intelligence-based pest counting method proposed in this study is suitable for constant and accurate monitoring of insect pests.

## 1. Introduction

The black pine bast scale, *Matsucoccus thunbergianae* (*Matsucoccus matsumurae*), is a major insect pest of black pine, *Pinus thunbergii*, in South Korea. The damage caused by *M. thunbergianae* to black pine was first reported on the southwest coast of the Korean Peninsula in 1963 [1]. Since then, the black pine bast scale has been dispersed throughout several coastal areas of the Korean peninsula [2]. Because of severe environmental and economic losses, intensive monitoring of the black pine bast scale has been conducted by the Korea Forest Service since 1983. The primary monitoring of the black pine bast scale involves surveying the egg sacs laid on pine twigs [2]. However, this method is time consuming, labor intensive, and requires skilled monitoring. Additionally, it is difficult to monitor egg sacs located in high places. Consequently, this method does not provide accurate monitoring data.

Pheromone traps have been widely used as valuable tools to monitor agricultural and forest insect pests worldwide. The sex pheromone emitted by the female black pine bast scale was first identified as matsuone, (2*E*,4*E*)-4,6-10,12-tetramethyl-2,4-tridecadiene-7-one [3,4], and Cywin et al. [5] determined the absolute configuration of matsuone as (2*E*,4*E*,6*R*, 10*R*)-4,6-10,12-tetramethyl-2,4-tridecadiene-7-one. In our previous studies [6,7], we developed a simple synthesis method for racemic matsuone and an effective trap to monitor the black pine bast scale for practical use in a field. The recognition and counting of insects in these pheromone traps have been mainly performed manually by humans. The process is labor intensive, and the results of the counting differ depending on the identification skill of the person [8]. In particular, for small-sized (1.5–2 mm) insects such as *M. thunbergianae*, there is a substantial difference in counting results because of identification skill and fatigue. To avoid labor-intensive manual counting, image-based automated monitoring methods have been used in many studies [9,10,11,12,13,14,15,16,17,18]. The image-based method captures images of traps and recognizes and counts pests on traps using image processing. Through this image-based method, the computing system undertakes the labor-intensive work, and it is also possible to automate counting immediately in the field using camera-trap systems without bringing the traps from the field into the lap. Further, there is no difference in counting due to identification skill and fatigue level, and the counting time can be shortened depending on the algorithm.

Recently, methods based on convolutional neural networks (CNNs) have shown high performance for tasks such as classification, detection, and segmentation in the field of vision [19,20,21]. CNNs are composed of layers such as convolution and pooling so that spatial information can be used in the network in a similar manner as filter methods in image processing. In object detection tasks, the proposed CNN-based object detectors show higher performance than conventional object detection methods. Region-based CNN (R-CNN), an early CNN-based object detection method, applies CNN to regions proposed in images, leading to performance improvement compared with existing machine learning-based methods [22]. Therefore, fast R-CNN with RoI pooling [23] and Faster R-CNN with RPN [24] have been proposed to improve performance and speed. After the two-stage object detectors of the R-CNN series, one-stage object detectors such as you only look once (YOLO) [25] and single-shot multibox detector (SSD) [26] have been developed and have reduced computing time compared with the R-CNN series. Recently, various CNN-based one-stage detectors have been developed, such as Retinanet [27] and EfficientDet [28].

These deep-learning-based object detection methods are applied to various vision-based detection tasks, such as pedestrian detection [29,30], object detection in aerial images [31,32,33], and object detection in agriculture [34,35,36]. Deep-learning-based object detection methods are also applied in aerial-image-based wild animal monitoring studies, similar to trap-based pest monitoring, and show higher performance than traditional image processing and machine learning methods [37,38,39,40]. Image-based studies for pest monitoring have used conventional image processing methods and machine learning classifiers [10,11,12,13]. However, recent studies based on deep learning have been increasing. Conventional image classification and detection methods require the human selection of image features, which has limitations compared to CNN-based methods that automatically learn suitable features according to the tasks. Ding et al. [14] applied the sliding window method and CNN to detect moths in trap images, and Nam et al. [15] applied an adaptive threshold and CNN, SSD, sliding window, and CNN methods to detect pests and compared the results. Nieuwenhuizen et al. [16] used Faster R-CNN to detect trapped insects, and Chulu et al. [17] identified and classified fall army worm moths based on CNN. In our previous study [18], we applied several deep learning object detection models, such as Faster R-CNN, R-FCN, Retinanet, and SSD to detect three types of moths in trap images and compared the speed and performance of each model.

Although these previous studies were conducted, studies related to the image-based detection of forest pests are insufficient when considering various types of forest pests, and studies on methods to improve the performance for detecting small-sized pests are also insufficient. To optimize the detection model, it is necessary to compare various object detection models and apply the latest detection models. Therefore, in this study, we applied deep learning counting methods to monitor *M. thunbergianae*. Image cropping was applied to successfully detect small-sized *M. thunbergianae*, and eight types of deep learning-based object detection models were trained, and their performances were evaluated. In addition, the counting accuracy according to each cropping condition and deep learning detector conditions was evaluated.

## 2. Materials and Methods

### 2.1. Data Collection

#### 2.1.1. Chemicals

The synthesis scheme of (6*R*,10*R/S*)-matsuone and the sex pheromone of the black pine bast scale was described in our previous paper [6]. The purity of the matsuone was above 97%. Butylated hydroxyltoluene (BHT, ≥99%) and hexane (98.5%) were purchased from Sigma-Aldrich (Milwaukee, WI, USA) and Daejung Chemicals & Metals Co., Ltd. (Siheung City, Gyeonggi-do, Korea), respectively. BHT was used as an antioxidant.

#### 2.1.2. Trap Collection

**Figure 1:**
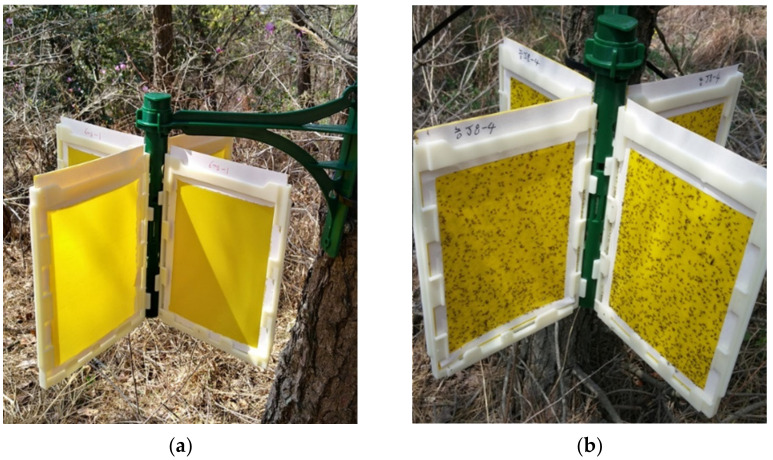
Eight-sided sticky trap: (**a**) installed pheromone traps; (**b**) captured *M. thunbergianae*.

Trap collection was conducted in a black pine stand in Gunsan (35°5755″ N, 126°3320″ E), Jeollabuk-do, Korea. Eight-sided sticky traps (Korea Institute of Pheromone, Daejeon City, Korea) were used for the field experiments (Figure 1). The trap consisted of four plates (130 mm width × 20 mm height), which were inserted into the cross vanes. Papers covering both yellow sticky sides (11 cm width × 15.5 cm height) of each plate were removed after the installation of the pheromone trap. Cable ties (8.8 mm width) were used to tie the frame of the trap to a pine tree stem. Matsuone (400 μg) and BHT (400 μg) were dissolved in hexane and loaded onto a rubber septum (Wheaton Scientific, Millville, NJ, USA). Pheromone traps were installed 50 cm above the ground on 25 March 2020. We collected the plates on 7 April 2020, and transferred them to the laboratory.

#### 2.1.3. Image Acquisition

**Figure 2:**
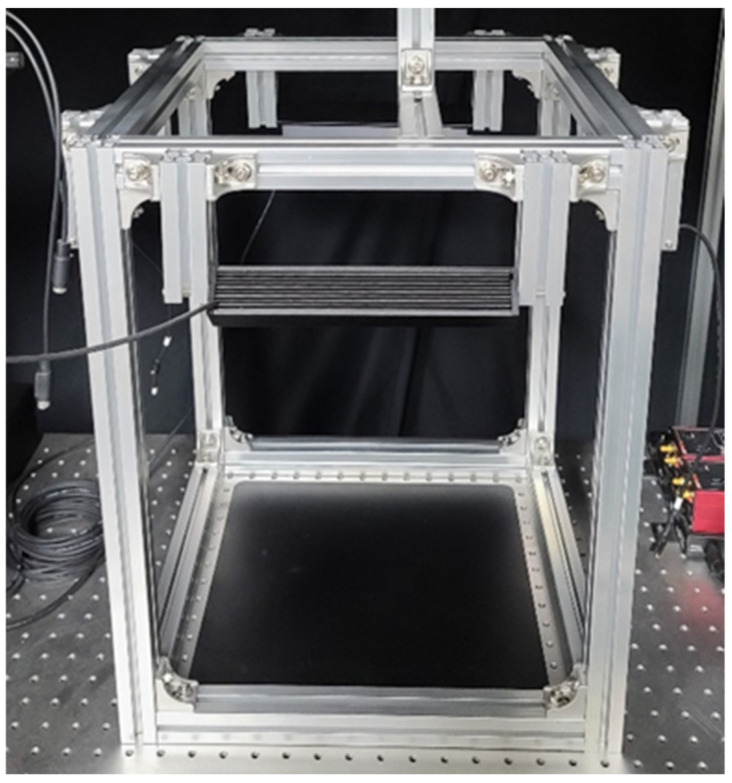
Trap photographing system.

**Figure 3:**
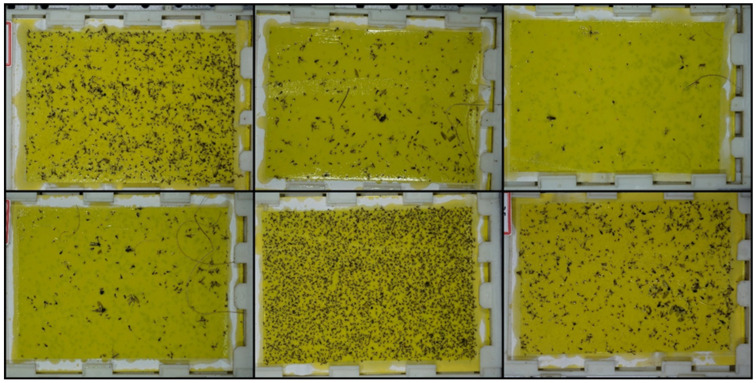
Samples of pheromone trap images.

The collected traps were photographed in a dark room using a photographing system with 4-way LED lighting, as shown in Figure 2. A color camera (α-6000, Sony Co., Tokyo, Japan) was attached to the top of the system, and photographs were taken at a resolution of 6000 × 4000 pixels. Figure 3 shows some of the trap images.

### 2.2. Data Preparation

For 50 of the collected trap images, annotations were performed for the four coordinates (xmin, ymin, xmax, ymax) of the ground truth bounding boxes of *M. thunbergianae*. In 50 images, 23,056 targets were annotated, and the size of the ground truth bounding box of *M. thunbergianae* was 60 × 60 pixels on average.

The annotated trap images were divided into a training set, a validation set, and a test set, as shown in Table 1. The training set was used for training the object detectors, the validation set was used for the evaluation of the detectors according to the parameter changes, and the test set was used for the performance evaluation of the optimized detectors.

### 2.3. Detector Training and Evaluation

**Figure 4:**
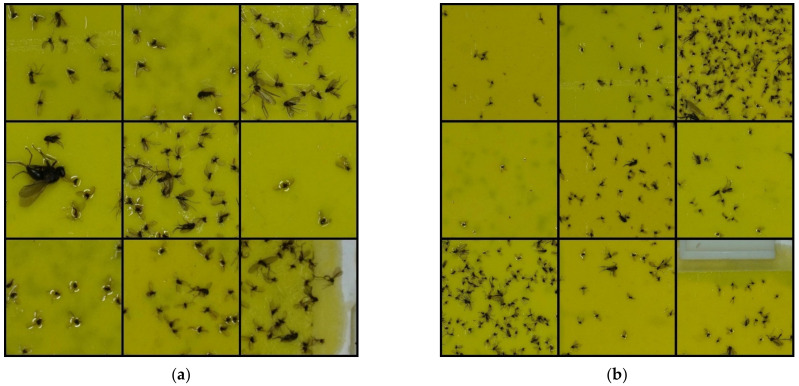
Cropped pheromone trap images: (**a**) 12 × 8 cropping condition; (**b**) 6 × 4 cropping condition.

Detecting small objects is a challenging task in object detectors, including deep-learning-based detectors. Compared with the image size, smaller objects result in less successful detection. In our trap image dataset, the resolution of images is 6000 × 4000 pixels, whereas the size of the *M. thunbergianae* is only 60 × 60 pixels on average, which can lead to a decrease in detection performance. One way to overcome this problem is to crop the image [40,41]. Because the cropped image has a larger object size relative to the image size than the uncropped image, the detection performance can be increased by cropping. Additionally, cropping and detecting images can increase object detection performance; however, it has the disadvantage of slowing down detection speed as detection must be performed multiple times. Considering this, to compare the detection speed and performance for cropping conditions, our detectors were trained by cropping the entire 6000 × 4000 images under the conditions of 12 × 8 and 6 × 4. Figure 4 shows cropped trap images with two different cropping conditions. A Quadro RTX-6000 GPU (Nvidia Corp., Santa Clara, CA, USA) was used for model training, validation, and testing.

Convolutional object detection models have differences in speed and accuracy depending on the configuration of the model [42]. Several factors affect the speed and accuracy, such as the type of meta-architecture, the type of feature extractor, and the input size. It is necessary to select and fine-tune a model that is suitable for the conditions required for the detection tasks. Therefore, in this study, we used 16 model conditions of four model types, two input sizes, and two cropping conditions. Model configurations according to model types and input sizes were Faster R-CNN Resnet 101, with input sizes of 1024 and 512; EfficientDet D0 and D4, which have input sizes of 512 and 1024, respectively; Retinanet 50, with input sizes 1024 and 640; and SSD MobileNet 2, with input sizes of 640 and 320. The code for the process was constructed based on the TensorFlow Object Detection API [42]. (The code constructed by this study will be copyrighted as software in the year 2021, and the algorithm will be available upon request after the copyright has been obtained). For the robustness of the model, transfer learning was applied using pretrained weights with the COCO dataset and data augmentation techniques such as vertical and horizontal flip, random crop and pad, contrast, and brightness adjustment. 

The trained models were evaluated using the average precision (AP) metric. The average precision is an evaluation metric widely used in object detection and is the average of the precision values according to the change in the recall value in the precision-recall distribution of the model. In the matching process between ground truth boxes and detected boxes, the acceptance of the box localization, considered as the correct detection, varies according to the intersection of union (IoU) threshold. The IoU is an indicator of how much the two boxes overlap and is calculated by dividing the overlap area by union areas. In this study, APs were calculated for IoU thresholds of 0.5 and 0.3. The reason for evaluating the threshold of 0.3 as well as the commonly used threshold of 0.5, is that the importance of accurate box localization is less than that of general object detection tasks in the case of counting purposes. The precision and recall used in the calculation of AP are given in Equations (1) and (2), where TP is the number of true positives, FP is the number of false positives, and FN is the number of false negatives.

(1) $$\mathrm{Precision}\;(\%)=\frac{\mathrm{TP}}{\mathrm{TP}+\mathrm{FP}}\times 100$$

(2) $$\mathrm{Recall}\;(\%)=\frac{\mathrm{TP}}{\mathrm{TP}+\mathrm{FN}}\times 100$$

### 2.4. Counting Accuracy Evaluation

Manual counting was performed on 30 traps to evaluate the counting accuracy. The 30 traps consisted of 10 traps each with less than 300, 300 to 500, and more than 500 *M. thunbergianae*. The counting was conducted by a person skilled in counting *M. thunbergianae* and was used as the basis for evaluating the counting accuracy of the detection model.

**Figure 5:**
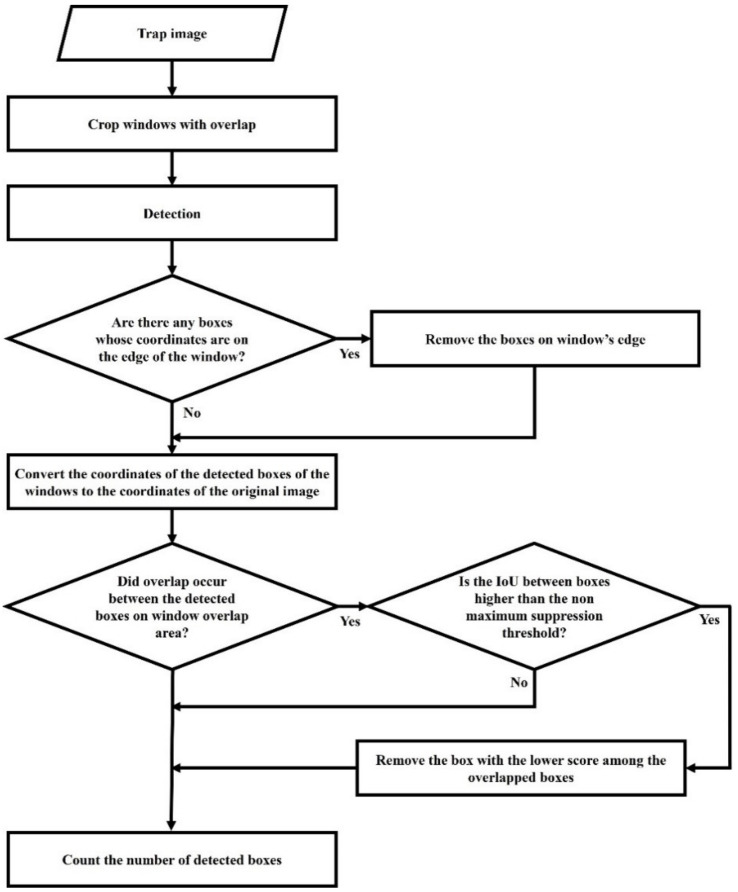
Flowchart for trap image counting.

The detection accuracy of the detector was evaluated on the original trap images (noncropped images), and for this purpose, the sliding window method was used. For each cropping condition, the original image was scanned and detected with a window of 500 × 500 for the 12 × 8 cropping condition and 1000 × 1000 for the 6 × 4 cropping condition. The overlap of the scanning was set to 100 pixels, which is larger than the size of *M. thunbergianae;* therefore, *M. thunbergianae* at the edge of the window could be completely contained inside the next window. Considering the overlap, the 6000 × 4000 trap images underwent 150 scans of 15 × 10 in the 12 × 8 cropping condition and 35 scans of 7 × 5 in the 6 × 4 cropping condition. Nonmaximum suppression (NMS) was performed to eliminate duplicate detections in overlapping areas. If an object is cut at the end of one window, only a part of the object is detected. In this case, the detection box has a low IoU with the same target’s detection box in the next window; therefore, duplicate detection is not removed by the NMS. To remove duplicate detections, when the box coordinates were located at the end of the window, the detection box was removed. Figure 5 shows a flowchart of our trap image counting algorithm.

**Figure 6:**
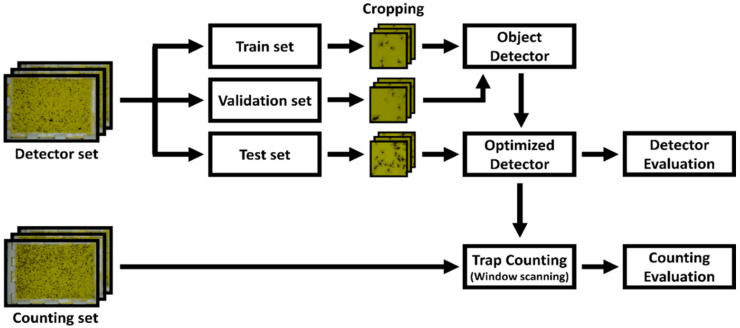
Schematic of the training and evaluation process.

The object detector can adjust the intensity of detection by adjusting the detection score threshold and the number of detected boxes changes according to this value. Therefore, 10 of the 30 images were used to determine the score threshold value for optimal counting for each model, and the counting accuracy was evaluated for the remaining 20 images based on the determined score threshold. The counting error was calculated using Equation (3).

(3) $$\mathrm{counting}\;\mathrm{error}\left(\%\right)=\overset{N}{\underset{i=1}{\sum }}\left|\frac{C_{i}-\hat{C_{i}}}{C_{i}}\right|\times 100$$

where $C_{i}$ is the number of manually counted *M. thunbergianae* in the ith trap, and $\hat{C_{i}}$ is the number of detected *M. thunbergianae* by the object detector in the ith trap. Figure 6 shows an overall schematic of the study.

## 3. Results

**Figure 7:**
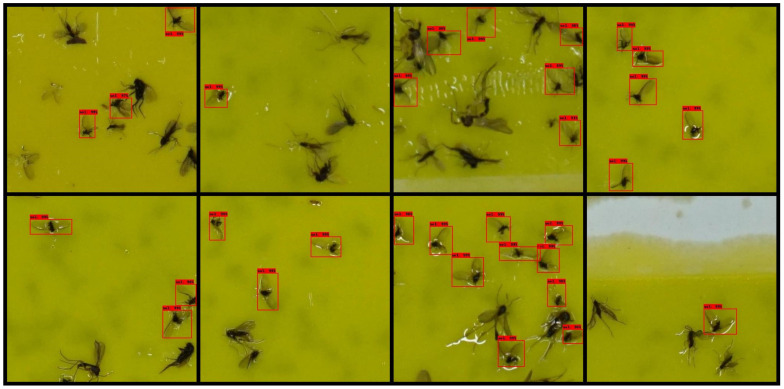
Detection result images of the 12 × 8 cropping condition (0.5 score threshold).

**Figure 8:**
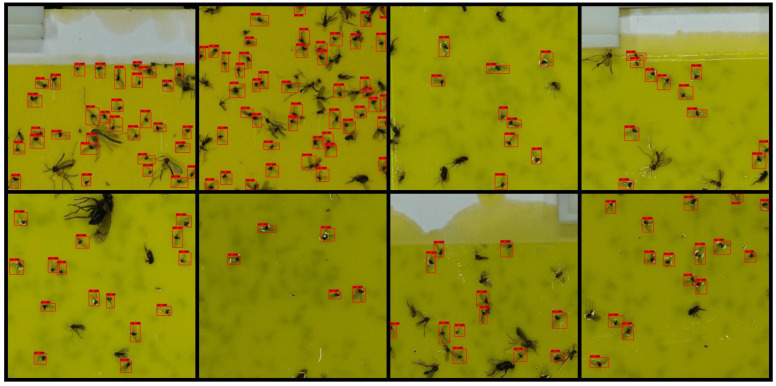
Detection result images of the 6 × 4 cropping condition (0.5 score threshold).

Table 2 and Table 3 list the test results of each model trained according to the 12 × 8 and 6 × 4 cropping conditions, respectively. Overall, the 12 × 8 crop-image-based detector resulted in higher AP. In the case of the 12 × 8 cropping condition, both the IoU thresholds of 0.5 and 0.3 resulted in similar APs regardless of the structure of the detection model. In the case of SSD MobileNet v.2 with a 320 input size, the inference time is seven times faster than that of Faster R-CNN Resnet 101 with a 1024 input size and EfficientDet D4 models, but APs were similar. This trend was similar to that of the models based on the 6 × 4 cropped image. Except for SSD MobileNet v.2 with a 320 input size, the AP difference was approximately 2–3% in both IoU thresholds 0.5 and 0.3 in the 6 × 4 cropping condition. In the case of SSD MobileNet v.2 with a 320 input size, APs were lower than those of the other models when applied on 6 × 4 cropped images by 5–7% at the IoU threshold of 0.5 and 8–11% at the IoU threshold of 0.3. Figure 7 and Figure 8 show the detection result image of Faster R-CNN Resnet 101 with a 1024 input size model under the 12 × 8 and 6 × 4 cropping conditions, respectively.

**Figure 9:**
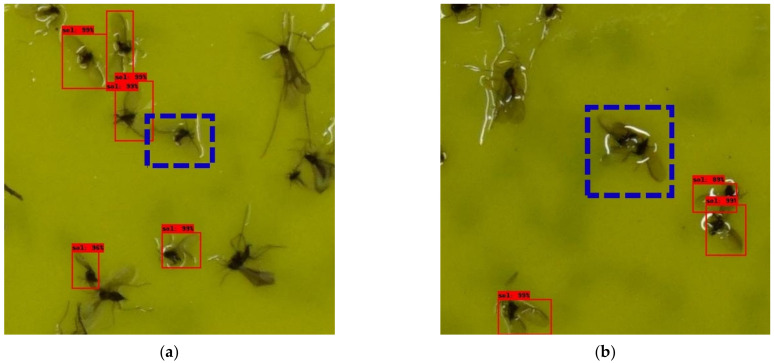

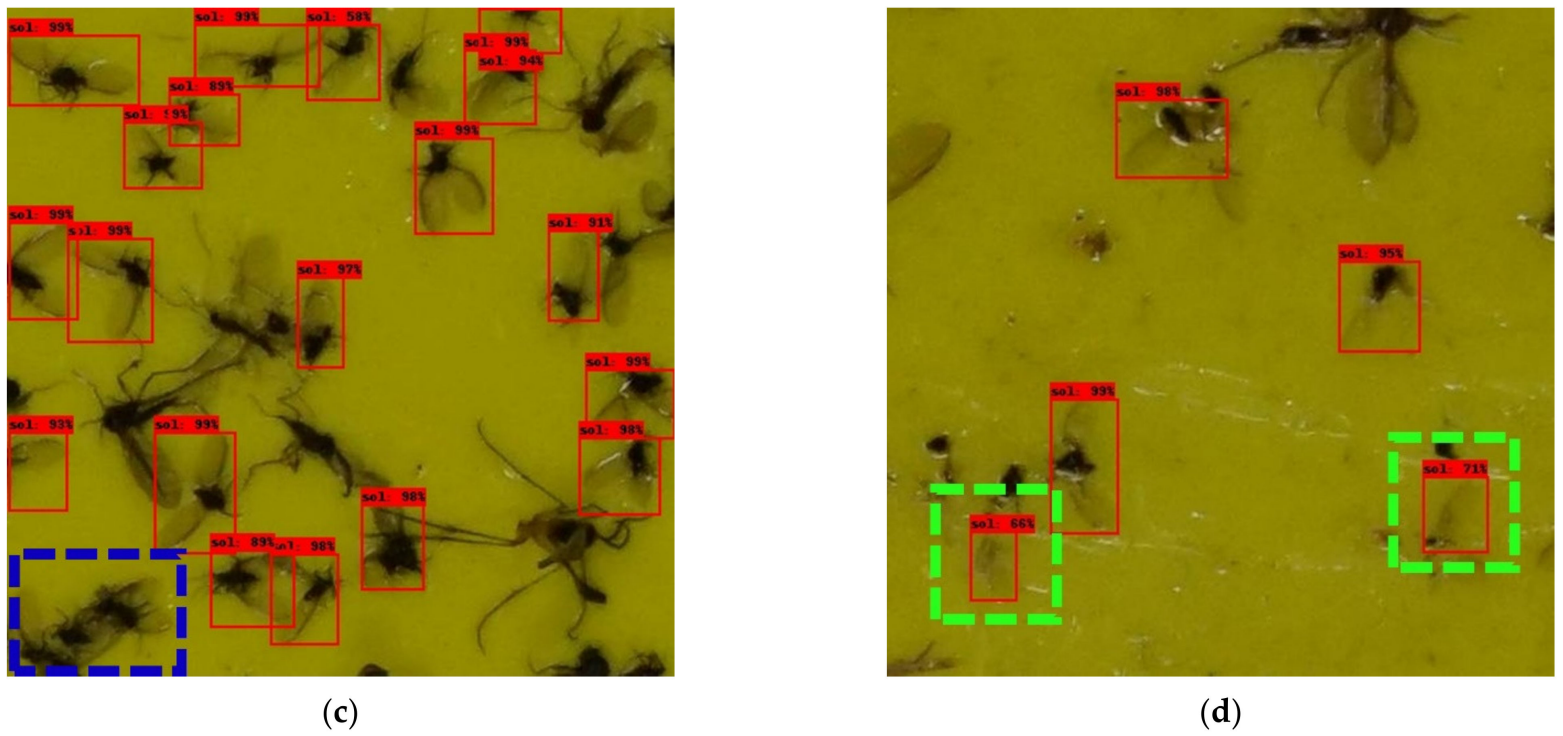
Detection errors: (**a**–**c**) false negatives; (**d**) false positives.

Figure 9 shows cases in which errors occurred during detection. Figure 9a–c shows error cases in which target objects were not detected. As shown in Figure 9b,c, when several insects were overlapped, these false negatives mainly occurred. There were many cases where it was difficult for even skilled researchers to distinguish whether it was *M. thunbergianae* or not because of overlapped insects, and most of the detection errors were in these cases. Figure 9d shows wings of insects that were mistakenly recognized as *M. thunbergianae*. These false-positive errors occurred less frequently when compared with false-negative errors. The types of errors in the 12 × 8 cropping condition and 6 × 4 cropping condition were similar and tended to occur more frequently in the 6 × 4 cropping condition.

**Figure 10:**
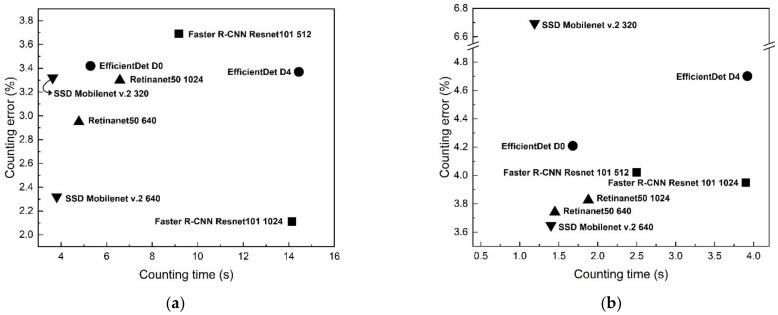
Counting error–counting time graph of detection models: (**a**) 12 × 8 cropping condition; (**b**) 6 × 4 cropping condition.

**Figure 11:**
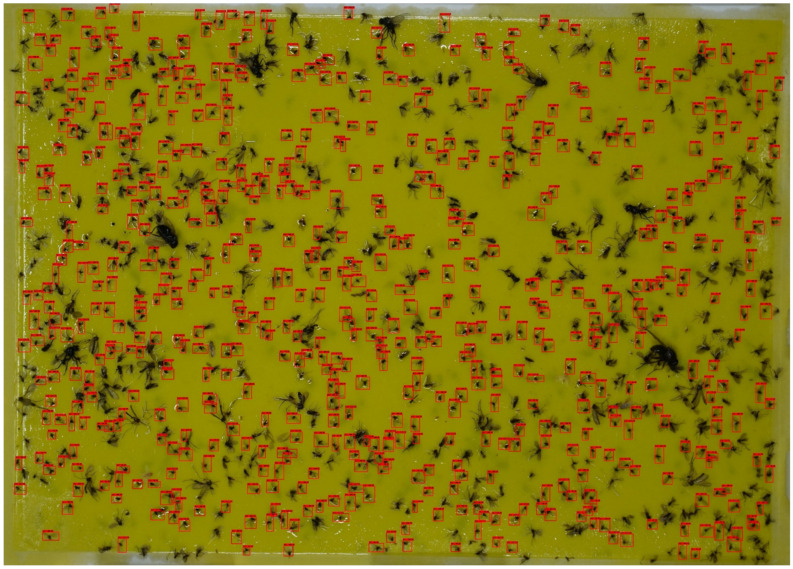
Detection results of the trap image (Faster R-CNN Resnet101 with a 1024 input size, a 12 × 8 cropping condition, and a 0.5 score threshold).

Figure 10 and Table 4 and Table 5 show the counting time and counting error regarding cropping conditions and model configurations. Similar to the detection evaluation results, the 12 × 8 cropping models showed a counting error of 2–3%, which is lower than the counting error of the 6 × 4 models, which was 3–9%. Among the 12 × 8 cropping models, Faster R-CNN Resnet101 with a 1024 input size showed the lowest counting error at 2.11%, and the other 12 × 8 cropping models showed counting errors of 2.35–3.69%. In the case of counting time, the 12 × 8 cropping models showed a range of 3.63–14.14 s, and the 6 × 4 cropping models showed a range of 1.19–3.92 s. This difference in counting time is because the 12 × 8 cropping model has 150 windows inspected during scanning, and the 6 × 4 model has 35 windows inspected. In the manual counting process, there were some differences in counting times according to the number of target insects included in the trap. The average counting times were 199, 198, and 501 s for each population range of less than 300, 300 to 500, and more than 500. These counting times are approximately 8–27 times longer than the counting time of Faster R-CNN Resnet 101 with a 1024 input size of the 12 × 8 cropping condition (14.14 s) and 85–276 times longer than the counting time of SSD MobileNet v.2 with a 640 input size of the 6 × 4 cropping condition (1.4 s). As in the detection accuracy evaluation, the counting errors of the models under the 6 × 4 cropping condition were similar except for SSD MobileNet v.2 with a 320 input size. SSD MobileNet v.2 with a 320 input size showed a counting error of 6.694%, which was 2–3% higher than that of the other 6 × 4 cropping condition models. Except for SSD MobileNet v.2 with a 320 input size under the 6 × 4 cropping conditions, all models in the two cropping conditions showed more than 95% counting accuracy. Figure 11 shows a trap image that was detected with our sliding window algorithm (Faster R-CNN Resnet101 with a 1024 input size of the 12 × 8 cropping conditions).

## 4. Discussion

In this study, we applied object detection methods based on deep learning for automated pest monitoring. In particular, for the successful detection of *M. thunbergianae*, which is a small-sized insect, a method of cropping was applied to the original image. Accordingly, detection and counting accuracy were evaluated for two cropping conditions and eight model conditions. As a result, it was confirmed that deep learning object detection methods can be successfully applied to monitor *M. thunbergianae*.

Our detection and counting performance results show that the fast models can perform similarly to the slow models when the number of cropping is sufficient (in the 12 × 8 cropping condition). When comparing the 12 × 8 cropping condition with the 6 × 4 cropping condition, the 12 × 8 cropping condition resulted in higher overall AP because the object size relative to the image size increased. In the case of the 6 × 4 cropping condition with the IoU threshold of 0.3, most of the models showed 84–85% APs, showing a small difference compared with the 12 × 8 cropping condition, which showed 89% APs, but the SSD MobileNet v.2 model with an input size of 320 showed a low AP of 79.87%. This is because the size of objects in the 6 × 4 cropping images was too small for the light model such as SSD MobileNet v.2 of a 320 input size. In the detection error cases, false-negative errors were more common than false-positive errors. Most of the errors occurred in cases where it was difficult to recognize *M. thunbergianae* manually because several insects overlapped or the shape of the insect was unclear. Although *M. thunbergianae* has various shapes and there are many other trapped insects similar in appearance to *M. thunbergianae*, most of the detected images showed successful detections.

The counting accuracy results using deep learning object detection methods showed a similar tendency to that of the detection performance evaluation. Overall, the 12 × 8 cropping condition showed a lower counting error than the 6 × 4 cropping condition, and SSD MobileNet v.2 with a 320 input size model under the 6 × 4 cropping condition showed a higher counting error than the other models. Except for SSD MobileNet v.2 with a 320 input size model, other models had 95.3–97.89% counting accuracy, demonstrating that deep learning-based object detectors can be successfully used to monitor the population of *M. thunbergianae*.

In this study, two cropping conditions (12 × 8, 6 × 4) were used, and it was confirmed that both the detection accuracy and counting accuracy of the 12 × 8 cropping condition were higher than those of the 6 × 4 cropping condition. However, as the number of windows increases with an increase in the number of croppings, the image processing takes longer; therefore, the detection speed decreases. Thus, it is necessary to determine the cropping condition and model considering the speed–accuracy tradeoff according to the computing system and the required speed. Although the 12 × 8 cropping condition showed a higher overall performance than the 6 × 4 cropping condition, the results of the 6 × 4 cropping condition also have high performance. Though excluded from the results of this study, when testing the 3 × 2 cropping results for some models, the APs of the 3 × 2 cropping condition were approximately 60–70%. Thus, when cropping is less than 6 × 4, it can be confirmed that the performance drops sharply, and it seems that somewhere between the 6 × 4 cropping condition and 3 × 2 cropping condition is the boundary line of this sharp drop.

Recently, there have been many studies related to pest detection based on deep learning [14,15,16,17,18]. The studies are different in factors such as target insect type, size, imaging conditions, number of classes, and evaluation metrics; therefore, it is difficult to compare quantitative accuracy between studies. In previous studies that evaluated the counting accuracy in a similar manner to this study, the counting accuracy was 90.86% [43] and 92.6% [44]. Our study differs from previous studies in that the results were compared using various types of deep learning object detection models, and the cropping method applied for the detection method was optimized for small insects. Through this approach, a high counting accuracy of 95.3–97.89% was obtained, and the results were presented considering the tradeoff between the speed and accuracy of the model. These counting accuracies are high values compared with human errors during the manual counting process.

The trap images in this study were taken using fixed lighting conditions in the laboratory, and these images were used for model training and evaluation. Therefore, images were taken in a much more organized environment than the actual outdoor environment, and the accuracy of the trained model may be reduced in the images of the actual environment. Additionally, in the case of the models applied in this study, especially one-stage models, there are structures to detect multiscale objects. In this study, the size of the target object relative to the image size is almost determined, and thus optimization through structural modification is possible. In consideration of this, some scale parameters were fine-tuned, but it seems that it is possible to optimize accuracy and speed through additional structural modifications.

In a subsequent study, to acquire models that can be applied in the field, it is necessary to add and train the data acquired from the actual field, and they should also be applied to the validation and test process. For this, research on the optimal environment, such as the configuration of the trap device in the field or the selection of the optimal camera system are also required. In this study, because a high-resolution camera was used, the performance of the model could be improved by cropping images. However, in the case of low-resolution images, the performance increase by cropping is likely to be small. Therefore, according to the specifications of the camera system, it is necessary to optimize the field of view and cropping conditions of the images. Moreover, it is necessary to optimize the model structure in consideration of the speed–accuracy required by the purpose and field environment.

## 5. Conclusions

To improve the labor-intensive and time-consuming shortcomings of manual counting for pheromone trap monitoring, this study developed counting algorithms from pheromone trap images for detecting *M. thunbergianae*. To detect *M. thunbergianae*, various deep learning object detection models were applied for conditions such as meta-architecture, feature extractor, and input size. Additionally, for the detection algorithm optimized for small objects, image cropping was applied, and the results of the models were compared. For model robustness, transfer learning and data augmentation were applied during training, and hyperparameters were fine-tuned through the validation process. After training, the detection and counting performances of the model were evaluated, and the speed and accuracy for each condition were obtained. As a result, the counting accuracy of *M. thunbergianae* was 97.89% in the highest model, which shows that the deep learning object detection model and image cropping method can be very effective in counting *M. thunbergianae*. In addition, because the target speed and accuracy of the model may differ depending on the type of task or computing system performance, the speed and accuracy of various conditions were also compared. The results of our study confirmed that constant monitoring can be achieved with accurate performance using the AI-based pest detection system.

## Figures and Tables

**Table 1:** Number of images and *M. thunbergianae* of datasets.

	Train Set	Validation Set	Test Set
Images	30	10	10
*M. thunbergianae*	13,419	5071	4566

**Table 2:** Test results of *M. thunbergianae* detectors (12 × 8 cropping condition).

Model	Input Size	Inference Time (ms)	AP (%)	
			IoU:0.3	IoU:0.5
Faster R-CNN Resnet 101	1024	78.26	89.78	85.63
Faster R-CNN Resnet 101	512	39.64	89.58	84.32
EfficientDet D4	1024	86.74	89.26	84.79
EfficientDet D0	512	25.58	88.36	83.79
Retinanet 50	1024	30.97	89.35	84.40
Retinanet 50	640	20.56	89.86	86.40
SSD Mobilenet v.2	640	15.28	89.02	84.76
SSD Mobilenet v.2	320	11.82	89.46	84.54

**Table 3:** Test results of *M. thunbergianae* detectors (6 × 4 cropping condition).

Model	Input Size	Inference Time (ms)	AP (%)	
			IoU:0.3	IoU:0.5
Faster R-CNN Resnet 101	1024	79.58	87.13	82.92
Faster R-CNN Resnet 101	512	41.48	85.04	80.18
EfficientDet D4	1024	90.33	84.87	81.22
EfficientDet D0	512	26.12	85.30	80.21
Retinanet 50	1024	33.52	86.58	82.62
Retinanet 50	640	21.85	85.33	81.71
SSD Mobilenet v.2	640	16.83	85.75	81.35
SSD Mobilenet v.2	320	12.22	79.87	72.05

**Table 4:** Counting error results of the 12 × 8 cropping condition.

Model	Input Size	Counting Time (s)	Counting Error (%)
Faster R-CNN Resnet 101	1024	14.14	2.11
Faster R-CNN Resnet 101	512	9.17	3.69
EfficientDet	1024	14.44	3.37
EfficientDet	512	5.29	3.42
Retinanet50	1024	6.58	3.30
Retinanet50	640	4.78	2.95
SSD Mobilenet v.2	640	3.81	2.32
SSD Mobilenet v.2	320	3.63	3.32

**Table 5:** Counting error results of the 6 × 4 cropping condition.

Model	Input Size	Counting Time (s)	Counting Error (%)
Faster R-CNN Resnet 101	1024	3.90	3.95
Faster R-CNN Resnet 101	512	2.50	4.02
EfficientDet	1024	3.92	4.70
EfficientDet	512	1.68	4.21
Retinanet50	1024	1.88	3.83
Retinanet50	640	1.45	3.74
SSD Mobilenet v.2	640	1.40	3.65
SSD Mobilenet v.2	320	1.19	6.69
